# Microalgae cultivation: closing the yield gap from laboratory to field scale

**DOI:** 10.3389/fbioe.2024.1359755

**Published:** 2024-02-14

**Authors:** Benoit Guieysse, Maxence Plouviez

**Affiliations:** ^1^ Massey Agrifood Digital Laboratory, Massey University, Palmerston North, New Zealand; ^2^ School of Agriculture and Environment, Massey University, Palmerston North, New Zealand

**Keywords:** microalgae, sustainability, economic feasibility, commercial experience, field productivity

## 1 Introduction

For more than 70 years, countless research programs have aimed to develop microalgae-based products and services such as foods and biofuels, wastewater treatment, and carbon sequestration (Borowitzka, 2013b; Craggs et al., 2014; Chen et al., 2019; Araújo et al., 2021). Unfortunately, and despite this research generating significant knowledge advances in microalgal biology, reactor design, and biomass processing, microalgae cultivation remains a nascent industry centered around a few high-value food applications (Plouviez et al., 2022). To understand why there is still such a gap between academic expectations and commercial realities, this opinion article briefly reviews the state of the art on commercial microalgae production and discusses the constraints limiting its industrial uptake. Noteworthy, this article neither intends to present a comprehensive review of research advances in the field nor does it challenges the tremendous potential of microalgae biotechnology. Instead, we seek to raise awareness of the gap between the current expectations and reality of microalgal cultivation at scale in order to better inform future investment in the field.

## 2 Current commercial microalgae applications

Despite significant research investments, only a few facilities for microalgae-based food production or wastewater treatment are currently in operation. Microalgae biotechnology is indeed difficult to implement at scale (Section 3.) and consequently the cost of microalgae biomass is (e.g. 2.83–315 USD·kg^−1^ according to Vázquez-Romero et al., 2022). The following section reviews the current state of the art of industrial microalgae applications at scale with a focus on photosynthetic cultivation.

### 2.1 Food

Microalgae have been commercially cultivated to produce food and high-value chemicals for decades. Yet, only a few microalgae species are being commercially produced with *Arthrospira platensis* (Spirulina), *Chlorella vulgaris*, *Dunaliella salina*, and *Haematococcus pluvialis* dominating the market (Borowitzka, 2018; Araújo et al., 2021). Microalgae food products are also typically more expensive than ‘conventional’ alternatives (e.g. retail value of 105–190 USD·kg^−1^ for spirulina powder from Cyanotech Co, one of the oldest companies on the market, Vieira Costa et al., 2019), due to the high costs of microalgae cultivation and processing (Barros et al., 2015; Supermaniam et al., 2019; Plouviez et al., 2022). Please see Belay, (1997); Borowitzka, (2013a); Grobbelaar, (2008); Olaizola, (2000) for further examples of commercial cultivation of microalgae for food production.

### 2.2 Wastewater treatment

Microalgae-based wastewater treatment in high rate algae ponds (HRAPs) is often heralded as a sustainable alternative to conventional wastewater treatment because algal photosynthesis reduces the energy demand for wastewater aeration and enables nutrient recovery in microalgae biomass (Craggs et al., 2012; Craggs et al., 2014; Alcántara et al., 2015). While the cost related to microalgae-based wastewater treatment in HRAPs have been projected to be similar to the cost of conventional wastewater treatment (0.15—0.25 USD·m^−3^, Posadas et al., 2017), only few demonstration-scale HRAPs have been tested and only a handful full-scale processes are currently in operation (Craggs et al., 2014; Supermaniam et al., 2019). A reason for this low implementation could be that the wastewater treatment industry is slow to adopt new technologies. Most likely, it is because of the prohibitive land requirement in urban environment (Roostaei and Zhang, 2017), the dependence of performance to environmental conditions (Craggs et al., 2012), or the fact the pollutant-laden biomass produced still represents a cost rather than a valuable product (Posadas et al., 2017).

### 2.3 Biofuel

Significant research is focusing on microalgae cultivation for bioenergy production (see references in Shuba and Kifle, 2018; Li et al., 2022). Yet, the costs of microalgae-based biofuels (e.g. USD 0.43 to 8.75 L^−1^ according to Quinn and Davis, 2015) remain prohibitively expensive compared to similar fuels and the sustainability of microalgal biofuels are nowadays increasingly challenged (Quinn and Davis, 2015; Park and Lee, 2016; Ketzer et al., 2017; Moshood et al., 2021). This poor performance likely explains why there is today no commercial microalgae biofuel facility in operation (Maeda et al., 2018; Chen et al., 2019) and why most of the companies that were once developing these commodities have either ceased to operate or pivoted towards the production of food and high value products (e.g., Algenol: https://www.algenol.com/).

### 2.4 Biorefinery

The concept of microalgal biorefineries is resurgent and often proposed as the best approach to maximize the economic and environmental value of microalgae cultivation (Pires et al., 2012; Vanthoor-Koopmans et al., 2013; Laurens et al., 2017; Park et al., 2022). For example, Beckstrom et al., 2020 concluded that a microalgae biorefinery using CO_2_-riched flue gas could economically and sustainably co-produce bioplastic feedstock and fuel. Unfortunately, there is currently no commercial microalgae-based biorefinery in operation.

## 3 Microalgae cultivation at large scale

Scale is needed to reduce the costs of nearly all industrial process. In the case of microalgal cultivation, the land area needed can be staggering, as further described in Section 3.3, but the largest microalgae farms currently under operation are only 36–44 ha in size (Plouviez et al., 2022), which highlights the lack of maturity of this industry. The following section briefly describes how microalgae can be cultivated at scale.

### 3.1 Process design and operation

Most microalgae commercial cultivation takes place in photobioreactors that improve control, productivity, quality, and biomass recovery. Various closed photobioreactors, such as column, tubular, and flat plate photobioreactors, have therefore been designed to optimize light utilization efficiency (especially by reducing light inhibition), gas-liquid mass transfer, and biomass harvesting (Borowiak et al., 2021; Cui et al., 2021; Yaqoubnejad et al., 2021; Wang et al., 2022b; Leong et al., 2023). However, and despite inherent risks of contamination, open raceways ponds remain the system of choice for commercial production as these systems are more economic to build and operate than closed designs (Jorquera et al., 2010; Craggs et al., 2012). Close systems are therefore hitherto only used to generate small amounts of biomass for research, very high-value applications, or the inoculation of larger raceways. This is best exemplified by the fact nearly all commercial spirulina growers use open ponds (Maeda et al., 2018; Araújo et al., 2021). Following cultivation, various processes can be used to harvest biomass, including centrifugation and filtration. Subsequent processing can include biomass drying (e.g., *Chlorella*, Spirulina) or extraction prior to drying (e.g., astaxanthin from *Haematococcus*). Please see Barros et al. (2015) for a comprehensive review on the topic.

Light supply, nutrients availability, pH, and temperature are key operational parameters to optimize microalgal growth. Nutrient supply and pH can be controlled during full scale operation via, for example, on-line pH monitoring and CO_2_ injection (Borowitzka and Vonshak, 2017). Unfortunately, controlling light supply and/or temperature is impractical or uneconomic in outdoor applications where, at best, a raceway pond can be located inside a greenhouse to enable some degree of climate control (Lu et al., 2011; Soni et al., 2017). This inability to properly control light supply and temperature causes productivity and biomass composition to fluctuate seasonally and geographically, and this variability prevents numerous high-value applications from being scalable and/or economic.

### 3.2 Climate and location

Locations with high solar radiations are forecasted to support high productivity but these climatic conditions are also typically associated with high water evaporation and high temperature-mediated biomass losses. For example, Béchet et al. (2014) and Béchet et al. (2016) demonstrated that growing *C. vulgaris* in an arid climate could cause severe local water stress and, without temperature control, would not increase annual biomass productivity due to the frequent cultures’ crashes caused by high temperature. Finally, prime locations with favorable climate and access to high-quality water are likely already used for conventional farming, meaning microalgae cultivation for low-value applications such as biofuel or ‘bulk’ food production will likely need to take place on ‘marginal’ or low-value agricultural land. The ability to grow microalgae on marginal land is arguably a critical advantage over conventional agriculture. Unfortunately, the use of such locations may exacerbate logistic challenges, as discussed in Section 3.4 below.

### 3.3 The need for scale

By linearly extrapolating the simulations from Plouviez et al. (2022), 200–280 ha of raceway ponds would theoretically be required to provide 10% of the recommended dietary intake of proteins of 1 million persons with microalgal biomass. In comparison, 600–1,200 ha would be needed to treat the domestic wastewater generated by this population, and 5,200–10,400 ha to supply 5%–10% of the fuel-energy required for domestic transportation using microalgae biodiesel. Without considering the space required to cultivate inoculum, process the biomass, and store chemicals and biomass, which can all together represent 10%–20% of the full operation (Borowitzka and Vonshak, 2017), this simple calculation highlights the scale required for microalgae biotechnology to truly have a global impacts in these markets: It will indeed be likely very difficult to find the land required to treat wastewater near the urban and sub-urban centers where this waste is produced and collected, and the land required to sustainably use the microalgae-rich biomass thus generated (Posadas et al., 2017). It is not all ‘gloom and doom’ however as from a global perspective, the land requirement for microalgae-based food production is relatively modest, and in fact similar to the land required by the most productive crops for a similar biomass output (e.g., 73–87 t·ha^−1^·yr^−1^ for sugarcane, Dismukes et al., 2008).

### 3.4 Other constraints

As with most industrial processes, scale is critical to improve the economics of microalgae cultivation and, typically, the minimal scale required for profitability increases when the value of the product or service sold decreases. This need for scale brings many additional constraints during commercial operation, such as local availability for energy, water, skilled manpower, nutrients, and CO_2_. Scale also increases local pressure to properly manage waste (including wastewater) and generates logistic challenges that can increase costs and risks, especially at remote locations. New challenges may also arise when considering multi-purposes refineries as the delivery of a product or service, such as wastewater treatment, may cause to over-supply and/or decrease the quality of other outputs. The need to cope with variability in inputs (such as light and temperature, but also wastewater composition and flow) can cause requirements for large design safety factors, storage, and standardization. Please consult Guieysse and Plouviez, (2021) and Plouviez et al. (2022) for a more comprehensive discussion.

## 4 Commercial microalgae cultivation: state of the art

The cultivation of microalgae to produce food products is currently the only established commercial microalgal biotechnology. Microalgae cultivation indeed remains difficult to scale-up and the non-biological constraints discussed above can cause considerable costs. Consequently, microalgal biotechnology is still immature and severely lacks a supporting industry that can provide the experience, services, and equipment required by new entrants. The resulting high costs of microalgae production means that low-value applications are currently not economically feasible, which in turn limits the development of microalgae biotechnology into a mature industry. The microalgae community is well aware of these challenges and several groups are developing harmonized frameworks and technical standards to boost the microalgal biotechnology industry (see Lane et al., 2021; Laurens, 2021).

### 4.1 Productivity yield gap

**FIGURE 1:**
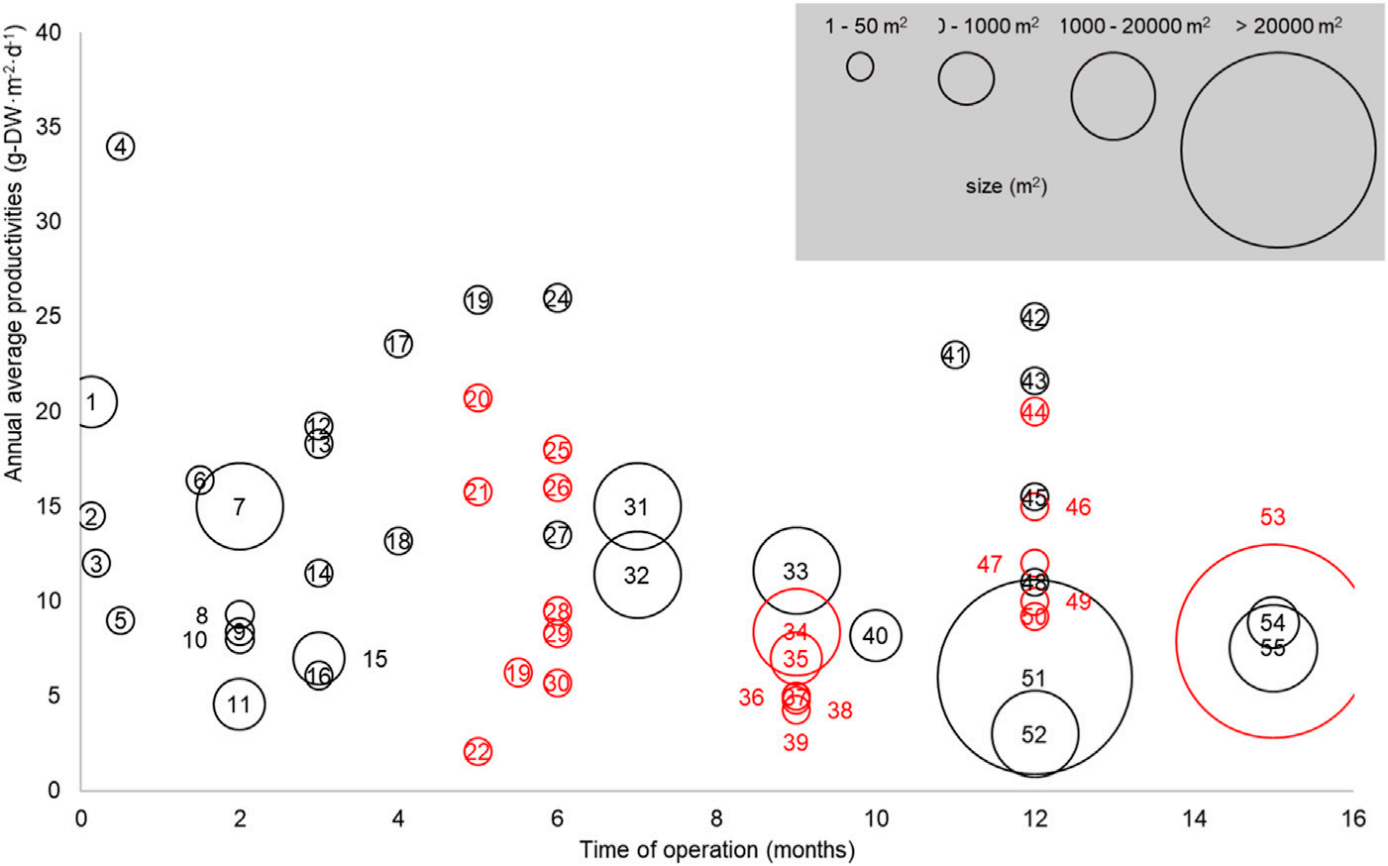
Productivities, duration of operation, and scale reported from 38 independent studies for microalgae cultivated in outdoor raceway ponds (black circles, g−DW·m^−2^·d^−1^) and high rate algal ponds (red circles, g−TSS·m^−2^·d^−1^) between 1987—2021 (adapted from Plouviez et al., 2022 with permission). Only data from raceways and high rate algal ponds are reported because these systems are the most suitable to grow microalgae at scale. See Supplementary Material S1 for further details about the data and the full references list.

Real-life productivity tends to decrease with the scale and duration of operation, which is illustrated in Figure 1 (see Supplementary Material S1 for references) summarizing experimental productivities recorded from 38 studies: As can be seen, productivities recorded during long-term (>12 months) and large-scale (>1,000 m^2^) trials average around 6 g−DW·m^−2^·d^−1^ and are significantly lower than the productivities recorded during short-term small-scale trials. This productivity yield gap between field and laboratory data is largely explained by the difficulty to economically maintain a high productivity under changing environmental conditions, as further discussed below.

### 4.2 Sustainability

Microalgae cultivation requires land, water, nutrients, and energy (for mixing and harvesting). Comparing data across studies is difficult due to the wide diversity of models and assumptions used during the theoretical assessments of costs and environmental impacts of microalgae biotechnologies, (Quinn and Davis, 2015; Guieysse and Plouviez, 2021; Roles et al., 2021). While several authors have suggested the need for harmonized assessments, microalgae cultivation and/or bioproduct generally appear to be a sustainable alternative to terrestrial crop farming in terms of land use and eutrophication risks (Guieysse and Plouviez, 2021). There is however a recurrent misconception that microalgae cultivation is intrinsically carbon negative because microalgae fix atmospheric CO_2_ into biomass during photosynthesis. Unfortunately, the CO_2_ assimilated during photosynthesis will be released back into the atmosphere (via respiration or combustion) shortly after its capture in most applications (Sander and Murthy, 2010), meaning that microalgae products are carbon neutral at best. In fact, various studies have shown that the carbon footprint of microalgae biotechnologies is positive, although the carbon footprint of microalgae products and services is generally lower than the footprints of ‘conventional’ alternatives (Lardon et al., 2009; Clarens et al., 2010; Roles et al., 2021).

## 5 Microalgae cultivation: how to close the productivity yield gap?

The following sections discuss the current development and considerations to reduce the gap between laboratory and field productivity yields.

### 5.1 Microalgae biology

Isolating and cultivating local species better adapted to local environmental conditions can improve stability (Olsen et al., 2021). Interestingly, little research has investigated the potential of seasonally rotating several strains or species although such approach could enable large gains in yearly productivities (Borowitzka, 2013c; Sun et al., 2020). The use of genetically modified strains could also help, although there are few reports of transformants with beneficial phenotypes and local regulations may limit practical implementation (Mosey et al., 2021; Plouviez et al., 2022). Independently of the strain(s) cultivated, daily management is critical to prevent biological contaminants (i.e., other algae, bacteria, virus and predators) that can rapidly overcome a culture. Please consult Borowitzka and Vonshak (2017) and Molina-Grima et al. (2021) for further details, and Harmon et al. (2021) for the use of specific metrics to quantitatively assess the impact of alien organisms on culture health and potential crashes.

### 5.2 Location

Local meteorological conditions are critical to optimize productivity and several models are available to estimate productivity at specific locations (Béchet et al., 2014; Béchet et al., 2016; Huesemann et al., 2018; Gao et al., 2022). However, as discussed above, location-dependent environmental and logistics constrains such as water supply, energy supply, biodiversity, manpower, transport, etc., must also be considered. Correa et al. (2019); Roles et al. (2021) provide good examples of approaches to evaluate the suitability of various locations to operate microalgae facilities.

### 5.3 Improving process design

Significant research is still ongoing to develop new reactor designs such as flat plate photobioreactor (Yaqoubnejad et al., 2021; Wang et al., 2022b) and automated modular airlift-type photobioreactor (Borowiak et al., 2021). In combination with modelling effort, reactors performance will also continue being improved (Gu et al., 2023). While the costs associated with construction and operation of advanced designs will always be high initially, their commercial adoption in high-value applications may gradually reduce their manufacture costs. While not directly relevant to increasing productivity, significant advances have also been achieved in biomass harvesting and processing, and this will certainly improve the overall process economics and environmental performance.

### 5.4 Improving process control

Because microalgal growth is highly dependent of the conditions experienced by the cells, it is critical that stresses are identified and controlled as soon as possible to maintain productivity and prevent culture crash (Borowitzka and Vonshak, 2017; Harmon et al., 2021; Mosey et al., 2021). The dynamic control of CO_2_ delivery and process parameters such as pond depth and hydraulic retention time can also be used to maximize productivity and minimize water stress (Béchet et al., 2016). On-line monitoring, possibly together with the use of the Internet of Things and machine learning, will enable development of smart technologies to automatically control operation (e.g., flow, depth, mixing, shading, CO_2_, pH, nutrient supply) based on, for example, environmental inputs and performance parameters (see Wang et al., 2022a for a review of this topic and Yan et al., 2021, for an example of how modelling could be used to inform operation real time).

### 5.5 Process integration

There is a consensus that in many applications, the cost of microalgae cultivation can only be afforded via the delivery of several products and services, such as combining biofuel feedstock production with wastewater treatment (Section 2.4.). As discussed above, there are some limitations to these approaches but one aspect that has not yet been well investigated is, to our opinion, how to best integrate microalgae production within the existing agri-food sector. Microalgae cultivation could indeed take advantage of food-safe waste streams generated by farms or processers, as well as unused processing capacity (e.g., drying) during periods of low farming outputs. Finally, looking at microalgae cultivation as a farming activity enables to budget land as an asset that appreciates over time rather than a cost that should be minimized. However, farmers must be able to confidently sell their bulk products to processers and/or wholesalers. Consequently, there is need to develop pathways to markets and rethink how to develop an algae industry that is well integrated within the entire value chain of the agri-food sector, as opposed to being developed in isolation.

### 5.6 Realistic expectations

**FIGURE 2:**
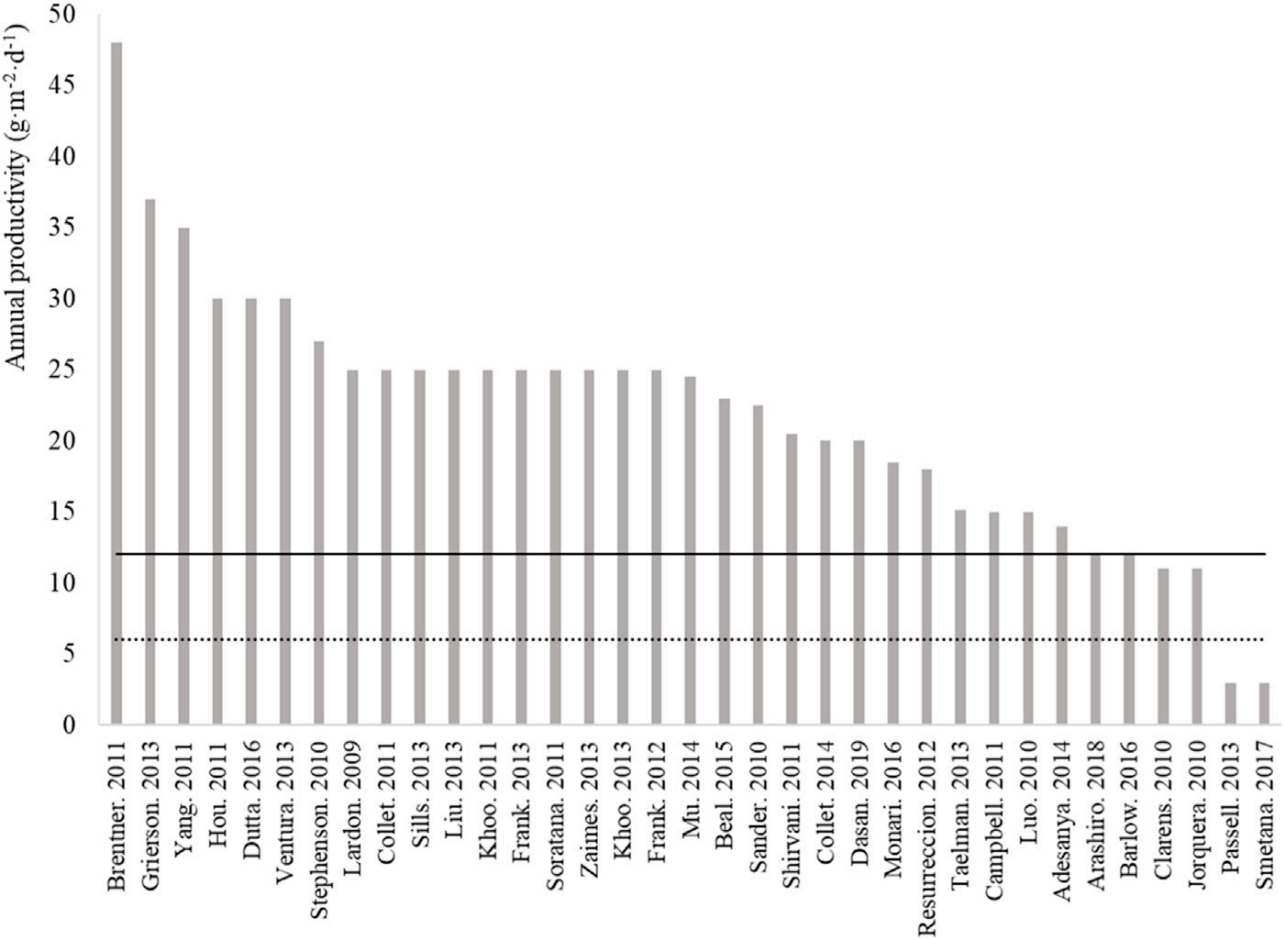
Base-case annual productivities used in some of the most cited life cycle assessment studies on microalgal biotechnologies (See Supplementary Material S2 for methodologies and references). The lower dotted lined represents the annual average productivity of 6 g−DW·m^−2^·d^−1^ calculated for the mature microalgae biotechnology industry (based on the annual productivities shown in Figure 1 for systems >1,000 m^2^ operated for at least 12 months, i.e., data n# 51–55). The plain line represent the expected annual average productivity for microalgae farming in the future defined as a doubling of current productivity based on experience from conventional crops (e.g., wheat productivity increased from 4.07 t·ha^−1^·yr^−1^ to 8.58 t·ha^−1^·yr^−1^ between 1964 and 2014 in the United Kingdom, Richie and Roser, 2013).

While the “laboratory-field yield gap” illustrated in Figure 1 is acknowledged in the literature (Plouviez et al., 2022), the data listed in Figure 2 evidence that most influential life cycle analyses of microalgae biotechnologies used productivity estimates far larger than the productivities currently achieved at commercial scale (i.e., ∼ 6 g−DW·m^−2^·d^−1^, Figure 1). Based on gains achieved with conventional crops between 1964 and 2014 (Ritchie and Roser, 2013), microalgae field productivity could double to 12 g−DW·m^−2^·d^−1^ with technological advances. This figure is similar to the annual biomass productivity of 15 g−DW·m^−2^·d^−1^ reported by Huesemann et al. (2018) when growing *Chlorella sorokiniana* in indoor raceway ponds under climate-simulated conditions. Yet, most assessments of microalgae cultivation are still based on over-optimistic estimates when considering these future gains, which may have caused poor investment choices. This must be corrected to optimize the allocation of research and commercial investments in the field.

## 6 Conclusion/recommendations

The commercial cultivation of microalgae remains complex and limited by numerous constraints. These challenges explain why despite decades of intense research, full-scale long-term productivities remain much lower than expectations from laboratory data and theoretical predictions. In the near future, higher field productivities will be achieved with new advances in strain selection (including rotation) and process improvement (design, operation, and control). Integration within existing supply chains will also be critical and has great potential for relatively high-value microalgae-to-food applications. Future investment should also seek to address the numerous constraints associated with microalgal production at scale.
