# Supplementary material for: Microalgae cultivation: closing the yield gap from laboratory to field scale
Source: Front Bioeng Biotechnol. 2024 Feb 14;12:1359755. doi: 10.3389/fbioe.2024.1359755 (PMC10901112; doi:10.3389/fbioe.2024.1359755)
Supplement: Supplementary file 1 [file DataSheet1.docx]

Supplementary Material

Microalgae cultivation: closing the yield gap from laboratory to field scale

Benoit Guieysse*, Maxence Plouviez

*** Correspondence:** Corresponding Author: B.J.Guieysse@massey.ac.nz

# Productivity data from the 38 studies reported in Figure 1

The numbers in bold are the legend for the numbers in Figure 1:

**1**: Weissman et al., 1988; **2**: Pushparaj et al., 1997; **3**: Huesemann et al., 2009; **4**: Ashokkumar and Rengasamy, 2012; **5**: Wang et al., 2018; **6**: Querijero-Palacpac et al., 1990; **7**: Grobbelaar, 2008; **8**: Saito et al., 2020 (average productivity obtained in 20 m^2^ raceway operated for 2 summer months); **9:** Saito et al., 2020 (average productivity obtained in 2 m^2^ raceway operated for 2 summer months); **10:** Saito et al., 2020 (average productivity obtained in 200 m^2^ raceway operated for 2 summer months); **11:** Saito et al., 2020 (average productivity obtained in 4000 m^2^ raceway operated for 2 months between summer and autumn);**12:** Boussiba et al., 1987 (average productivity from summer months); **13**: Vonshak and Guy, 1992 (productivity reported for Spirulina strain SP-G); **14**: Vonshak and Guy, 1992 (productivity reported Spirulina strain SP-RB); **15**: Koley et al., 2019; **16**: Boussiba et al., 1987 (average productivity from winter months); **17**: Laws et al., 1988; **18**: Hase et al., 2000; **19**: Sing et al., 2014; **20**: Park and Craggs, 2010 (productivity reported from a HRAP operated at 4 days HRT with CO_2_ addition); **21**: Park and Craggs, 2010 (productivity reported from a HRAP operated at 8 days HRT with CO_2_ addition); **22**: Posadas et al., 2015; **23**: Arbib et al., 2013; **24**: Richmond et al., 1990 (average productivity recorded between May-October); **25**: Cromar et al., 1996; **26**: Matamoros et al., 2015 (productivity reported from a HRAP operated at 4 days HRT); **27**: Richmond et al., 1990 (average productivity recorded between November - April); **28**: Matamoros et al., 2015 (productivity reported from a HRAP operated at 8 days HRT); **29**: Plouviez et al., 2019 (productivity reported from a HRAP operated at 7.5 days HRT); **30**: Plouviez et al., 2019 (productivity reported a HRAP operated at 10 days HRT); **31**: White and Ryan, 2015 (average productivity recorded between April – October in 2012); **32**: White and Ryan, 2015 (average productivity recorded between April – October in 2014); **33**: Lewis, 2015; **34**: Sutherland et al., 2020; **35**: Sutherland et al., 2020; **36**: Sutherland et al., 2014 (productivity reported from a HRAP with a culture depth of 20 cm); **37**: Sutherland et al., 2014 (productivity reported a HRAP with a culture depth of 30 cm); **38**: Sutherland et al., 2014 (productivity reported from a HRAP with a culture depth of 40 cm); **39**: Sutherland et al., 2020; **40**: Jimenez et al., 2003; **41**: AI-Shayji et al., 1994; **42**: Moheimani and Borowitzka, 2006 (average productivity recorded for *Pleurochrysis carterae*); **43**: Moheimani and Borowitzka, 2006 (average productivity recorded for *Dunaliella salina*); **44**: Arashiro et al., 2019 (productivity recorded from a HRAP receiving primary wastewater); **45**: Moreno et al., 2003; **46**: Arashiro et al., 2019 (productivity recorded from a HRAP receiving secondary wastewater); **47**: Marin et al., 2018; **48**: Matsumoto et al., 1995; **49**: Passos et al., 2015; **50**: Park and Craggs, 2013; **51**: Average productivity reported in 1991 by Belay, 1997; **52**: Passell et al., 2013; **53**: Craggs et al., 2012; **54**: Hong et al., 2016; **55**: Hong et al., 2017.

# Description of the Scopus search and full references list for Figure 2.

To list the biomass productivities commonly used during economics and sustainability assessments of microalgal biotechnology, we searched for the scientific literature focusing on those assessments. We identified this literature by searching for “microalgae”, “algae”, “algal”, “microalgal”, and “life cycle” within article titles in Scopus as followed:

TITLE ("microalgae" OR "algae" OR "microalgal" OR "algal") AND TITLE ("life cycle")).

This search, conducted on August the 1st 2023, yielded 380 documents cited 17334 times. Reviews, meta data analyses and studies clearly irrelevant to the purpose of our analysis were removed from the list. Overall, 241 documents all together cited 13067 times were kept. We then focused on the 43 most cited studies representing 70% of the total 13067 citations for the 241 selected studies. Productivity data was only available for 35 studies out of 43, but these 35 studies alone yielded 68% of the total citation of the 241 selected studies. Only the base case scenario productivity data were selected. Most of the 35 studies were focusing on virtual facilities cultivating microalgae in raceways ponds or HRAPs. If several cultivation designs were compared, only the data documented for raceways were selected. The data is presented in Figure 2 of the manuscript.

**References**

Adesanya, V. O., Cadena, E., Scott, S. A., and Smith, A. G. (2014). Life cycle assessment on microalgal biodiesel production using a hybrid cultivation system. *Bioresour Technol* 163, 343-55.

Al-Shayji, Y.A., Puskas, K. Al-Daher, R. (1994). Production and separation of algae in a high-rate ponds system. *Enviro International* 4, 541-550.

Arashiro, L. T., Ferrer, I., Rousseau, D. P. L., Van Hulle, S. W. H., and Garfi, M. (2019). The effect of primary treatment of wastewater in high rate algal pond systems: Biomass and bioenergy recovery. *Bioresour Technol* **280**, 27-36.

Arashiro, L. T., Montero, N., Ferrer, I., Acien, F. G., Gomez, C., and Garfi, M. (2018). Life cycle assessment of high rate algal ponds for wastewater treatment and resource recovery. *Sci Total Environ* 622-623, 1118-1130.

Arbib, Z., Ruiz, J., Álvarez-Díaz, P., Garrido-Pérez, C., Barragan, J., Perales, J.A. (2013). Long term outdoor operation of a tubular airlift pilot photobioreactor and a high rate algal pond as tertiary treatment of urban wastewater. *Ecol Eng* 52, 143-153.

Ashokkumar, V. Rengasamy, R. (2012). Mass culture of Botryococcus braunii Kutz. under open raceway pond for biofuel production. *Bioresour Technol* 104, 394-399.

Barlow, J., Sims, R. C., and Quinn, J. C. (2016). Techno-economic and life-cycle assessment of an attached growth algal biorefinery. *Bioresour Technol* 220, 360-368.

Beal, C. M., Gerber, L. N., Sills, D. L., Huntley, M. E., Machesky, S. C., Walsh, M. J., Tester, J. W., Archibald, I., Granados, J., and Greene, C. H. (2015). Algal biofuel production for fuels and feed in a 100-ha facility: A comprehensive techno-economic analysis and life cycle assessment. *Algal Res* 10, 266-279.

Belay, A. (1997). Mass culture of spirulina outdoors - The Earthrise Farms Experience in Vonshak, A. (ed), Spirulina platensis (Arthorspira): Physiology, cell biology and biotechnology., London, Taylor and Francis pp 131- 158.

Boussiba, S., Vonshak, A., Cohen, Z., Avissar, Y. Richmond, A. (1987). Lipid and biomass production by the halotolerant microalga *Nannochloropsis salina*. *Biomass* 12, 37-47.

Brentner, L. B., Eckelman, M. J., and Zimmerman, J. B. (2011). Combinatorial life cycle assessment to inform process design of industrial production of algal biodiesel. *Environ Sci Technol* 45, 7060-7.

Campbell, P. K., Beer, T., and Batten, D. (2011). Life cycle assessment of biodiesel production from microalgae in ponds. *Bioresour Technol* 102, 50-6.

Clarens, A. F., Resurreccion, E. P., White, M. A., and Colosi, L. M. (2010). Environmental Life Cycle Comparison of Algae to Other Bioenergy Feedstocks. *Environ Sci Technol* 44, 1813–1819.

Collet, P., Helias, A., Lardon, L., Ras, M., Goy, R. A., and Steyer, J. P. (2011). Life-cycle assessment of microalgae culture coupled to biogas production. *Bioresour Technol* 102, 207-14.

Collet, P., Lardon, L., Hélias, A., Bricout, S., Lombaert-Valot, I., Perrier, B., Lépine, O., Steyer, J.-P., and Bernard, O. (2014). Biodiesel from microalgae – Life cycle assessment and recommendations for potential improvements. *Renewable Energy* 71, 525-533.

Craggs, R., Sutherland, D., Campbell, H. (2012). Hectare-scale demonstration of high rate algal ponds for enhanced wastewater treatment and biofuel production. *J Appl Phyco* 24(3), 329-337.

Cromar, N.J., Fallowfield, H.J., Martin, N.J. (1996). Influence of environmental parameters on biomass production and nutrient removal in a high rate algal pond operated by continuous culture. *Wat Sci Technol* 34, 133-140.

Dasan, Y. K., Lam, M. K., Yusup, S., Lim, J. W., and Lee, K. T. (2019). Life cycle evaluation of microalgae biofuels production: Effect of cultivation system on energy, carbon emission and cost balance analysis. *Sci Total Environ* 688, 112-128.

Dutta, S., Neto, F., and Coelho, M. C. (2016). Microalgae biofuels: A comparative study on techno-economic analysis & life-cycle assessment*. Algal Res* 20, 44-52.

Frank, E. D., Elgowainy, A., Han, J., and Wang, Z. (2012a). Life cycle comparison of hydrothermal liquefaction and lipid extraction pathways to renewable diesel from algae. *Mitig Adapt Strat Glob Chang* 18, 137-158.

Frank, E. D., Han, J., Palou-Rivera, I., Elgowainy, A., and Wang, M. Q. (2012b). Methane and nitrous oxide emissions affect the life-cycle analysis of algal biofuels*. Environ Res Let* 7.

Grierson, S., Strezov, V., and Bengtsson, J. (2013). Life cycle assessment of a microalgae biomass cultivation, bio-oil extraction and pyrolysis processing regime. *Algal Res* 2, 299-311.

Grobbelaar, J.U. (2009). From laboratory to commercial production: a case study of a Spirulina (Arthrospira) facility in Musina, South Africa. *J Appl Phyco* 21(5), 523-527.

Hase, R., Oikawa, H., Sasao, C., Morita, M., Watanabe, Y. (2000). Photosynthetic production of microalgal biomass in a raceway system under greenhouse conditions in Sendai city. *J Biosci Bioeng* 89, 157-163.

Hong, J.W., Kim, O.H., Jo, S.W., Do, J.M., Yoon, H.S. (2017). Microalgal biomass productivity and dominant species transition in a Korean mass cultivation system. *Algal Res*, 26, 365-370.

Hong, J.W., Kim, O.H., Kim, H., Jo, S.W., Cho, H.W., Yoon, H.S. (2016). Mass cultivation from a korean raceway pond system of indigenous microalgae as potential biofuel feedstock. *Oil Gas Res* 2:108.

Hou, J., Zhang, P., Yuan, X., and Zheng, Y. (2011). Life cycle assessment of biodiesel from soybean, jatropha and microalgae in China conditions. *Renew Sust En Rev* 15, 5081-5091.

Huesemann, M.H., Hausmann, T.S., Bartha, R., Aksoy, M., Weissman, J.C. Benemann, J.R. (2009. Biomass productivities in wild type and pigment mutant of Cyclotella sp. (Diatom). *Appl Biochem Biotechnol* 157(3), 507-526.

Jimenez, C., Cossio, B.R., Labella, D., Niell, F.X. (2003). The Feasibility of industrial production of Spirulina (Arthrospira) in Southern Spain. *Aquaculture* 217, 179-190.

Jorquera, O., Kiperstok, A., Sales, E. A., Embirucu, M., and Ghirardi, M. L. (2010). Comparative energy life-cycle analyses of microalgal biomass production in open ponds and photobioreactors. *Bioresour Technol* 101, 1406-13.

Khoo, H. H., Koh, C. Y., Shaik, M. S., and Sharratt, P. N. (2013). Bioenergy co-products derived from microalgae biomass via thermochemical conversion--life cycle energy balances and CO_2_ emissions. *Bioresour Technol* 143, 298-307.

Khoo, H. H., Sharratt, P. N., Das, P., Balasubramanian, R. K., Naraharisetti, P. K., and Shaik, S. (2011). Life cycle energy and CO_2_ analysis of microalgae-to-biodiesel: preliminary results and comparisons. *Bioresour Technol* 102, 5800-7.

Koley, S., Mathimani, T., Bagchi, S.K., Sonkar, S., Mallick, N. (2019). Microalgal biodiesel production at outdoor open and polyhouse raceway pond cultivations: A case study with *Scenedesmus accuminatus* using low-cost farm fertilizer medium. *Biomass Bioenerg* 120, 156-165.

Lardon, L., Helias, A., Sialve, B., Steyer, J., and Bernard, O. (2009). Life-Cycle Assessment of Biodiesel Production from Microalgae. *Environ Sci Technol* 43, 6475-6481.

Laws, E.A., Taguchi, S., Hirata, J., Pang, L. (1988). Optimization of microalgal production in a shallow outdoor flume. *Biotechnol Bioeng* 31, 140-147.

Lewis, D. (2015). Project report: Advancing established and integrated marine microalgae biofuel to commercialisation, Muradel Pty Ltd.

Liu, X., Saydah, B., Eranki, P., Colosi, L. M., Greg Mitchell, B., Rhodes, J., and Clarens, A. F. (2013). Pilot-scale data provide enhanced estimates of the life cycle energy and emissions profile of algae biofuels produced via hydrothermal liquefaction. *Bioresour Technol* 148, 163-71.

Luo, D., Hu, Z., Choi, D. G., Thomas, V. M., Realf, M. J., Chance, R. R., and (2010). ife Cycle Energy and Greenhouse Gas Emissions for an Ethanol Production Process Based on Blue-Green Algae. *Environ Sci Technol* 44, 8670–8677.

Marin, D., Posadas, E., Cano, P., Perez, V., Lebrero, R., Munoz, R. (2018). Influence of the seasonal variation of environmental conditions on biogas upgrading in an outdoors pilot scale high rate algal pond. *Bioresour Technol* 255, 354-358.

Matamoros, V., Gutierrez, R., Ferrer, I., Garcia, J., Bayona, J.M. (2015). Capability of microalgae-based wastewater treatment systems to remove emerging organic contaminants: a pilot-scale study. *J Hazard Mater* 288, 34-42.

Matsumoto, H., Shioji, N., Hamasaki, A., Ikuta, Y., Fukuda, Y., Sato, M., Endo, N., Tsukamoto, T. (1995). Carbon dioxide fixation by microalgae photosynthesis using actual flue gas discharged from a boiler. *Appl bioch Biot* 51/52, 681-692.

Moheimani, N.R., Borowitzka, M.A. (2006). The long-term culture of the coccolithophore Pleurochrysis carterae (Haptophyta) in outdoor raceway ponds. *J appl Phycol* 18(6), 703-712.

Monari, C., Righi, S., and Olsen, S. I. (2016). Greenhouse gas emissions and energy balance of biodiesel production from microalgae cultivated in photobioreactors in Denmark: a life-cycle modeling. *J Clean Prod* 112, 4084-4092.

Moreno, J.E. (2013). Demonstration and deployment successes Sapphire integrated algal biorefinery, Presentation at Biomass 2013.

Mu, D., Min, M., Krohn, B., Mullins, K. A., Ruan, R., and Hill, J. (2014). Life cycle environmental impacts of wastewater-based algal biofuels. *Environ Sci Technol* 48, 11696-704.

Park, J.B., Craggs, R.J. (2010). Wastewater treatment and algal production in high rate algal ponds with carbon dioxide addition. *Water Sci Technol* 61(3), 633-639.

Park, J.B., Craggs, R.J., Shilton, A.N. (2013). Enhancing biomass energy yield from pilot-scale high rate algal ponds with recycling. *Water Res* 47(13), 4422-4432.

Passell, H., Dhaliwal, H., Reno, M., Wu, B., Ben Amotz, A., Ivry, E., Gay, M., Czartoski, T., Laurin, L., Ayer, N. (2013). Algae biodiesel life cycle assessment using current commercial data. *J Environ Manage* 129, 103-111.

Passos, F., Gutiérrez, R., Brockmann, D., Steyer, J.-P., García, J., Ferrer, I. (2015). Microalgae production in wastewater treatment systems, anaerobic digestion and modelling using ADM1. *Algal Res* 10, 55-63.

Plouviez, M., Chambonnière, P., Shilton, A., Packer, M.A., Guieysse, B. (2019). Nitrous oxide (N_2_O) emissions during real domestic wastewater treatment in an outdoor pilot-scale high rate algae pond. Algal Res 44 e101670

Posadas, E., Muñoz, A., García-González, M.-C., Muñoz, R., García-Encina, P.A. (2015). A case study of a pilot high rate algal pond for the treatment of fish farm and domestic wastewaters. *J Chemical Technol Biot* 90(6), 1094-1101.

Pushparaj, B., Pelosi, E., Tredici, M.R., Pinzani, E. Materassi, R. (1997). An integrated culture system for outdoor production of microalgae and cyanobacteria. *J Appl Phycol* 9, 113-119.

Querijero-Palacpac, N.M., Martinez, M.R., Boussiba, S. (1990). Mass cultivation of the nitrogen-fixing cyanobacterium *Gloeotrichia natans*, indigenous to rice-fields. *J Appl Phycol* 2, 319-325.

Resurreccion, E. P., Colosi, L. M., White, M. A., and Clarens, A. F. (2012). Comparison of algae cultivation methods for bioenergy production using a combined life cycle assessment and life cycle costing approach. *Bioresour Technol* 126, 298-306.

Richmond, A., Lichtenberg, B., Stahl, B., Vonshak, A. (1990). Quantitative assessment of the major limitations on productivity of Spirulina platensis in open raceways. *J Appl Phycol* 2, 195-206.

Saito, T., Ichibara, T., Inoue, H., Uematsu, T., Hamada, S., Watanabe, T., Takimura, Y., Webb, J. (2020). Comparison of Areal Productivity of *Nannochloropsis oceanica* Between Lab-Scale and Industrial-Scale Raceway Pond. *Mar Biotechnol*. 22, 836-841.

Sander, K., and Murthy, G. S. (2010). Life cycle analysis of algae biodiesel. The International *J Life Cycl Assess* 15, 704-714.

Shirvani, T., Yan, X., Inderwildi, O. R., Edwards, P. P., and King, D. A. (2011). Life cycle energy and greenhouse gas analysis for algae-derived biodiesel. *En Enviro Sci* 4.

Sills, D. L., Paramita, V., Franke, M. J., Johnson, M. C., Akabas, T. M., Greene, C. H., and Tester, J. W. (2013). Quantitative uncertainty analysis of Life Cycle Assessment for algal biofuel production. *Environ Sci Technol* 47, 687-94.

Sing, F.S., Isdepsky, A., Borowitzka, M.A., Lewis, D.M. (2014). Pilot-scale continuous recycling of growth medium for the mass culture of a halotolerant *Tetraselmis sp.* in raceway ponds under increasing salinity: a novel protocol for commercial microalgal biomass production. *Bioresour Technol* 161, 47-54.

Smetana, S., Sandmann, M., Rohn, S., Pleissner, D., and Heinz, V. (2017). Autotrophic and heterotrophic microalgae and cyanobacteria cultivation for food and feed: life cycle assessment. *Bioresour Technol* 245, 162-170.

Soratana, K., and Landis, A. E. (2011). Evaluating industrial symbiosis and algae cultivation from a life cycle perspective. *Bioresour Technol* 102, 6892-901.

Stephenson, A. L., Kazamia, E., Dennis, J. S., Howe, C. J., Scott, S. A., and Smith, A. G. (2010). Life-Cycle Assessment of Potential Algal Biodiesel Production in the United Kingdom: A Comparison of Raceways and Air-Lift Tubular Bioreactors. *En Fuels* 24, 4062-4077.

Sutherland, D.L., Park, J., Heubeck, S., Ralph, P.J., Craggs, R.J. (2020). Size matters – Microalgae production and nutrient removal in wastewater treatment high rate algal ponds of three different sizes. *Algal Res* 45.

Sutherland, D.L., Turnbull, M.H., Craggs, R.J. (2014). Increased pond depth improves algal productivity and nutrient removal in wastewater treatment high rate algal ponds. *Water Res* 53, 271-281.

Taelman, S. E., De Meester, S., Roef, L., Michiels, M., and Dewulf, J. (2013). The environmental sustainability of microalgae as feed for aquaculture: a life cycle perspective. *Bioresour Technol* 150, 513-22.

Ventura, J. R., Yang, B., Lee, Y. W., Lee, K., and Jahng, D. (2013). Life cycle analyses of CO2, energy, and cost for four different routes of microalgal bioenergy conversion. *Bioresour Technol* 137, 302-10.

Vonshak, A., Guy, R. (1992). Photoadaptation, photoinhibition and productivity in the bluegreen alga, Spirulina platensis grown outdoors. *Plant, Cell and Enviro*. 15, 613-616.

Wang, Z., Wen, X., Xu, Y., Ding, Y., Geng, Y. Li, Y. 2018. Maximizing CO_2_ biofixation and lipid productivity of oleaginous microalga *Graesiella sp.* WBG-1 via CO_2_-regulated pH in indoor and outdoor open reactors. *Sci Total Environ* 619, 827-833.

Weissman, J.C., Goebel, R.P. Benemann, J.R. (1988) Photobioreactor design: mixing, carbon utilization, and oxygen accumulation. *Biotechnol Bioen* 31, 336-344.

White, R.L., Ryan, R.A. (2015). Long-Term Cultivation of algae in open-raceway ponds: Lessons from the field. *Industrial Biotechnology* 11(4), 213-220.

Yang, J., Xu, M., Zhang, X., Hu, Q., Sommerfeld, M., and Chen, Y. (2011). Life-cycle analysis on biodiesel production from microalgae: water footprint and nutrients balance. *Bioresour Technol* 102, 159-65.

Zaimes, G. G., and Khanna, V. (2013). Microalgal biomass production pathways: evaluation of life cycle environmental impacts. *Biotech Biofuels* 6, 1-11.
